# Finger Flexion to Noxious Stimulation in a Brain-dead Patient: A Case Report and Review of Literature

**DOI:** 10.7759/cureus.3622

**Published:** 2018-11-22

**Authors:** Zalan Khan, Christopher R Newey, Pravin George, Lary Raber

**Affiliations:** 1 Neurology, University of Missouri, Columbia, USA; 2 Neurology, Cleveland Clinic, Cleveland, USA

**Keywords:** abnormal movements, hoffmann's reflex, brain dead, finger flexion

## Abstract

Several guidelines and definitions for brain death have been proposed. The Uniform Determination of Death Act (UDDA) in 1980, the American Academy of Neurology (AAN) guidelines in 1995 and the later update in 2010 have all described standards for diagnosing brain death. As brain death testing became more commonly performed, several abnormal reflexive movements were recognized and led to ambiguities that falsely suggested retained brain function. Movements in the upper extremities have been under-recognized. We report a brain-dead patient with finger flexion in the upper extremities with noxious stimulation and suspect a pathogenesis similar to that of Hoffman's reflex sign. We present a case of an asthmatic patient who presented with pulseless electrical activity (PEA). The patient was managed emergently and subsequently deteriorated to a comatose state. She remained in a comatose state following management and showed diffuse cerebral edema secondary to anoxic brain injury on computed tomography (CT) scan. Subsequent apnea testing, transcranial Doppler studies (TCD) and detailed neurological examinations were performed. She was eventually declared brain dead. On nailbed pressure to her fourth finger, she had flexion of her third finger, similar to the finding of a Hoffman’s sign in an upper motor neuron injury. We have described this case in detail and reviewed the literature on abnormal movements in brain-dead patients.

## Introduction

The legal definition of death, as described by the Uniform Determination of Death Act (UDDA) of 1980, includes irreversible cessation of circulatory and respiratory functions, termed “cardiac death”, or irreversible cessation of all functions of the entire brain, including the brain stem also called “brain death” [1]. Medical standard to evaluate a person as brain dead was further formalized by the American Academy of Neurology (AAN) in 1995. It emphasized three key clinical findings: coma due to a known cause, absence of brainstem reflexes, and apnea [2].

Despite these guidelines, variability among leading academic institutions remained [3,4]. The AAN updated brain death guidelines in 2010 considering several factors that previously led to ambiguities such as ancillary testing, addressing confounders, and complex motor movements that falsely suggest retained brain function [3].

As brain death testing became more commonly performed, additional abnormal reflexive movements were recognized. These include subtle ocular micro-tremors to obvious limb reflexes (such as triple flexion in the lower extremities) or of the torso (the “Lazarus” sign) [5]. Movements in the upper extremities have largely been under-recognized. Here, we report a brain-dead patient with finger flexion in the upper extremities with noxious stimulation. We suspect the movements to be similar to the pathological Hoffman’s reflex sign.

## Case presentation

A 41-year-old female with a past medical history significant for asthma presented to an outside hospital emergency department in pulseless electrical activity (PEA) arrest. Her husband found her earlier in the morning on the couch with nebulizer treatment in hand. She quickly progressed to being unresponsive and cardiopulmonary resuscitation (CPR) was started. When emergency medical service (EMS) arrived (approximately 10 minutes from the onset of CPR), she was in PEA arrest. She was given two doses of epinephrine. Return of spontaneous circulation (ROSC) was achieved. She was taken to an outside hospital (OSH).

She was hypotensive (50/41 mmHg) requiring vasopressor infusion and had diffuse expiratory wheezing requiring continuous nebulizer treatment. Her initial arterial blood gas (ABG) was significant for a pH of < 6.8, carbon dioxide (CO_2_) of 130 mmHg, and oxygen (O_2_) of 331 mmHg. After continuous albuterol treatments and adjustments to the ventilator, a repeat ABG had the following values: pH of 6.81, PaCO_2_ of 138 mmHg, and PaO_2_ of 262 mmHg. On examination, she remained comatose with fixed and dilated pupils (6 mm, nonreactive) with a Glasgow Coma Scale (GCS) score of 3T. She was transferred to our facility for consideration of extracorporeal membrane oxygenation (ECMO).

**Figure 1:**
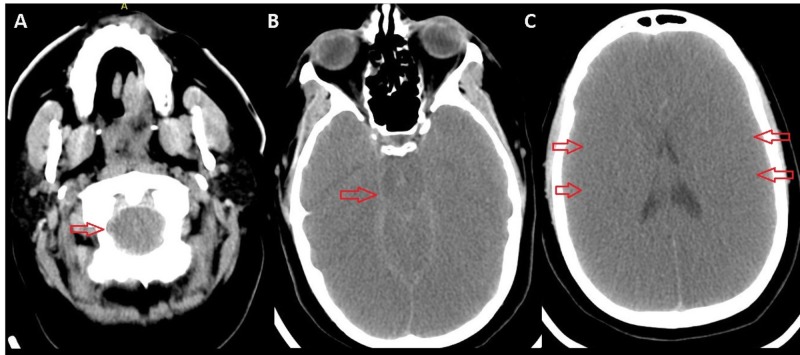
Computed tomography (CT) of the head. Diffuse cerebral edema is noted with tonsillar herniation (A), uncal herniation (B), and loss of cortical sulci (C).

On arrival, she was sedated and paralyzed to optimize ventilation/oxygenation prior to ECMO. Computed tomography (CT) of the head showed diffuse cerebral edema concerning for severe anoxic brain injury (Figure 1). She was evaluated by the neurologic intensive care unit (NICU) team. She was given mannitol (100 g) and 23.4% (30 cc) without a change in the neurological examination. Veno-venous (V-V) ECMO was started (flow 4 LPM, speed 3215 RPM, FiO_2_ of 100%, sweep 9 L/min, with ventilator FiO_2_ of 40%). Her ABG improved to pH of 7.29 and PaCO_2_ of 36. She was then transitioned from epinephrine to norepinephrine.

Over the next 12 hours, she developed polyuria (7.2 L) with increasing sodium (from 147 meq/L to 172 meq/L). She was given desmopressin and started on a D5W infusion. Her sodium improved to 149 meq/L over the next 12 hours. Her ABG now had a pH of 7.37, PaCO_2_ of 39 mmHg, and PaO_2_ of 90 mmHg. Continuous electroencephalography (CEEG) showed background suppression. Despite the correction of severe acid-base disorder and electrolyte disorders, she remained in a comatose state with 6 mm, nonreactive pupils. She was noted to have absent oculocephalic, oculovestibular, cough, gag, and not triggering ventilator. There was no change in heart rate with noxious stimulation. Apnea test was attempted. She was pre-oxygenated. She was then disconnected from respirator with six liters of oxygen (O_2_) delivered to carina via red rubber suction tubing. The sweep on the ECMO was adjusted to 1 L/min. However, she quickly desaturated to 82% for >30 seconds. The test was aborted. She was reconnected to the ventilator and ECMO adjusted. A second attempt occurred after pre-oxygenation. The sweep this time was reduced to 4 L/min from 9 L/min. She again did not tolerate, and the test was aborted. She was reconnected to the respirator. ECMO was changed back to sweep of 9 L/min. Her temperature was 36.2°C at the time of testing. Transcranial Doppler (TCD) was then ordered.

**Figure 2:**
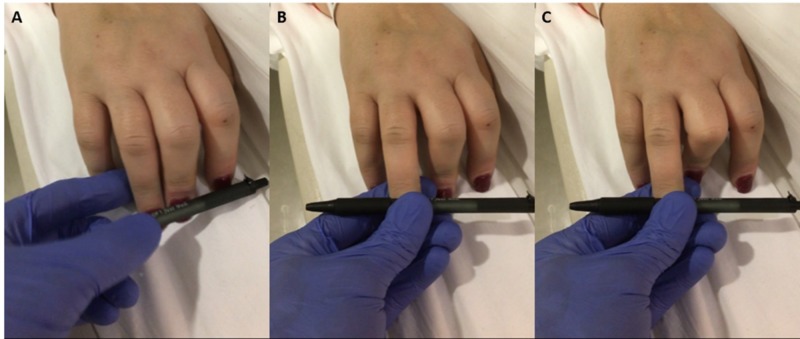
Finger movements. (A) Noxious pressure applied to the fourth fingernail bed. (B) As nail bed pressure is applied, the third finger begins to flex. (C) Final flexion of the third finger is noted after one second of nail bed pressure.

**Figure 3:**
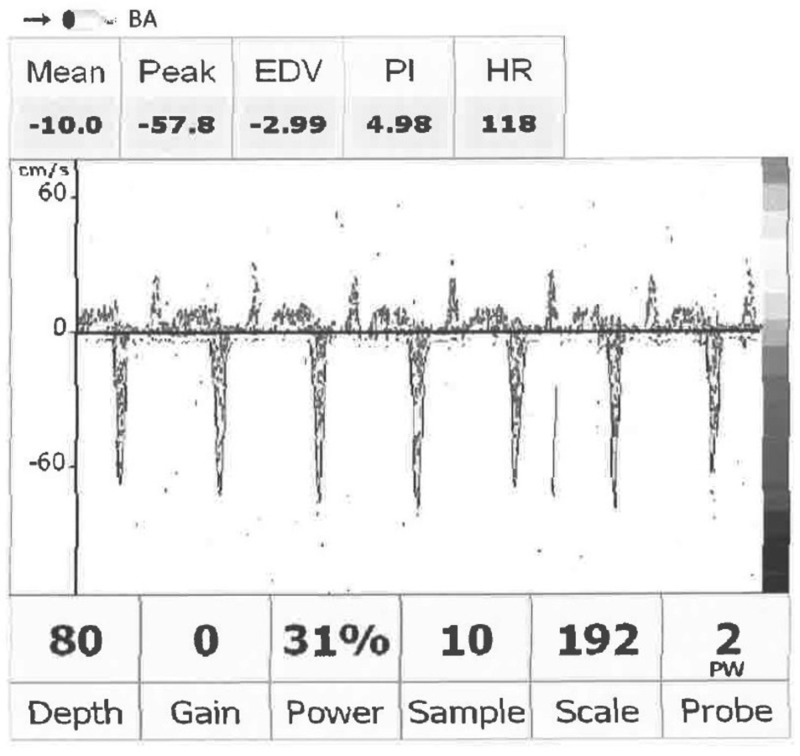
Transcranial Doppler (TCD). Illustrative image showing systolic spikes in the basilar artery. Arteries insolated in the anterior circulation showed similar findings.

The following morning while awaiting TCD to be performed, repeat neurological examination again found her to be in a coma with brainstem areflexia, except for performing apnea test. On nailbed pressure to her fourth finger, she had flexion of her third finger – similar to the finding of a Hoffman’s sign in an upper motor neuron injury (Figure 2). This flexion occurred over one second. Her lower extremities remained areflexic and flaccid. She was noted to have reduced ECMO requirements. The decision was made to repeat the apnea test. Her ABG had a pH of 7.39, PaCO_2_ of 37 mmHg, and PaO_2_ of 138 mmHg. Again, she was prepared by pre-oxygenating and then disconnected from the respirator. A cannula was placed in the endotracheal tube (ETT) and delivered six liters of O_2_ to the carina. The sweep on the ECMO was systematically decreased over the five-minute period down to 500 cc/min. She tolerated this adjustment. Her O_2_ saturation remained 95–100% during repeat apnea test. After 37 minutes of testing, her ABG showed a pH of 7.2, PaCO_2_ of 60 mmHg, and PaO_2_ of 147 mmHg. She was declared brain dead. TCD was eventually completed and interpreted as systolic spikes (Figure 3).

## Discussion

Our case report highlights an unusual finding of upper extremity finger flexion with noxious stimulation. We suspect this movement to be similar to the Hoffman’s reflex sign seen in patients with upper motor neuron injury.

Movements occurring in brain death are well-documented and notorious for invalidating or delaying definitive diagnosis of brain death [6]. Recent reviews categorize brain-death associated movements into stimulus-mediated reflexes and spontaneous automatisms with a hypothesized spinal mechanism. Irrespective of the nature of these movements, the majority are observed within the first 72 hours of diagnosis of brain death with the earliest showing almost immediately and the latest up to six days later [7].

Ivan described his findings in 52 brain-dead patients, of whom, 35% had muscle stretch reflexes, 60% had a plantar reflex response, 75% had abdominal reflexes and plantar withdrawal was observed in 35% [8]. This was followed by Jorgensen who noted seven different reflexes in 79% of 63 brain-dead patients and described unilateral extension-pronation movements of the upper limb in response to a cutaneous stimulus [9]. Spittler et al. and Dösemeci et al. described similar reflexes with larger cohorts of 235 and 134, respectively [10,11]. It is important to point out that Spittler et al. also categorized movements in brain-dead patients as spinal reflex patterns and automatism patterns. Additionally, they explain that abnormal movements can occur in up to two-thirds of brain-dead patients but may not be recognized due to their subtleness [10].

Saposnik et al. described several different movements in 38 brain-dead patients ranging from subtle spontaneous jerks of the fingers to the more pronounced “Lazarus sign” which causes raising of the arms and then dropping them on the chest [12]. The group then went on to prospectively analyze a multicenter cohort of 107 brain-dead patients for undulating toe flexion movements (UTF) as a common spinal reflex seen in up to 23% of cases [13]. Conci et al. noted contractions of the abdominal musculature in 60% of their cohort of 25 brain-dead patients during donor nephrectomy following the formal declaration of brain death [14]. Some movements in brain-dead patients can be subtler and/or fleeting such as transient bilateral symmetrical flexion-extension movements of all fingers [15], repetitive leg movements [16], facial myokymia, and flaring of alae nasi [12] and thus require keen observation. Hoffman-like reflexive movements have not been reported related to brain death.

The exact pathophysiology of abnormal movements in brain-dead patients remains complex, yet evidence points towards the spinal cord as the apparent source [7]. Saposnik et al. proposed that the undulating toe flexion movements observed in their cohort were spinal in origin as somatosensory evoked potentials (SSEP) did not produce any cortical responses [13]. Conci et al. reported findings of muscular contracture and changes in blood pressure and heart rate in response to peritoneal manipulation as spinal viscero-somatic and viscero-visceral reflexes, respectively [14]. Interesting to note, Schneider and Matakas observed spinal reflexes in brain-dead patients where ischemic lesions were above C1-C4 pointing to the hypothesis of a cut-off point between a disconnected and therefore a disinhibited spinal cord as the origin [17].

Spittler et al. gave special focus to the phenomenological diversity and underlying neuroanatomical considerations when evaluating spinal reflexes in brain-dead patients. Furthermore, the team categorized the movements into proprioceptive reflexes, exteroceptive reflexes and automatisms without recognizable triggering stimulus based on hypothesized spinal anatomical localizations [10].

De Freitas et al. explain complex spinal reflexes in brain-dead patients while performing TCD studies and their association with hypotension and mechanical stimulation. Individuals exhibiting the movements showed vertebrobasilar circulatory arrest [18]. De Freitas and Andre later noted that 55% of their brain-dead patients’ cohort retained plantar reflexes but none had Babinski sign and associated the movement pattern to the triple flexion response consisting of flexion at the hip and knee, and dorsiflexion of the foot [19]. Han et al. describe medullary hypoxia and hypercapnia-induced activity of cervical cord neurons, lack of central inhibition of movement generators of the spinal cord, and mechanical irritation of spinal cord due to neck flexion/extension leading to abnormal movements as possible reasons for abnormal movements in their cohort of 26 brain-dead patients [20].

Despite the multiple reports on abnormal movements in brain-dead patients, none report finger flexion to noxious stimulation. In light of current literature [7], we would categorize the finger-flexion in our patient as a stimulus-provoked reflex rather than a spontaneous automatism. Furthermore, we did not note any rhythmic-tremor-like repetitive movements as noted by Araullo et al. or any movements in the lower limb [15]. We hypothesize a mechanism similar to that of the Hoffmann’s reflex involving the Rexed lamina IX of the spinal cord coupled with an increased excitability and disinhibition of corticospinal and rubrospinal tracts responsible for distal limb control. We understand the actual pathophysiology to be more complex and therefore encourage more detailed studies. We consider our finding to be compatible with brain death and therefore highlight it.

## Conclusions

The conundrum of definitively diagnosing brain death in patients, in spite of existing guidelines, is well documented. Abnormal movements in such patients further add to the diagnostic complexity. We report a rare case of finger-flexion in response to a noxious stimulus in light of the limited existing literature to further the understanding of such complex movements. We hypothesize a pathophysiology similar to that of the Hoffmann's reflex and consider the findings compatible with brain death. Furthermore, we encourage more detailed studies involving larger cohorts of such patients to fully understand the actual pathophysiology.

## References

[1] Spinello IM (2013). Brain death determination. J Intensive Care Med.

[2] Wijdicks EF, Varelas PN, Gronseth GS, Greer DM (2010). Evidence-based guideline update: determining brain death in adults: report of the Quality Standards Subcommittee of the American Academy of Neurology. Neurology.

[3] Citerio G, Crippa IA, Bronco A, Vargiolu A, Smith M (2014). Variability in brain death determination in Europe: looking for a solution. Neurocrit Care.

[4] Greer DM, Varelas PN, Haque S, Wijdicks EF (2008). Variability of brain death determination guidelines in leading US neurologic institutions. Neurology.

[5] Busl KM, Greer DM (2009). Pitfalls in the diagnosis of brain death. Neurocrit Care.

[6] Plum F, Posner JB (1982). The Diagnosis of Stupor and Coma. https://global.oup.com/academic/product/plum-and-posners-diagnosis-of-stupor-and-coma-9780195321319?cc=ca&lang=en&.

[7] Jain S, DeGeorgia M (2005). Brain death-associated reflexes and automatisms. Neurocrit Care.

[8] Ivan LP (1973). Spinal reflexes in cerebral death. Neurology.

[9] Jorgensen EO (1973). Spinal man after brain death. The unilateral extension-pronation reflex of the upper limb as an indication of brain death. Acta Neurochir.

[10] Spittler JF, Wortmann D, von During M, Gehlen W (2000). Phenomenological diversity of spinal reflexes in brain death. Eur J Neurol.

[11] Dosemeci L, Cengiz M, Yilmaz M, Ramazanoglu A (2004). Frequency of spinal reflex movements in brain-dead patients. Transplant Proc.

[12] Saposnik G, Maurino J, Bueri J (2001). Movements in brain death. Eur J Neurol.

[13] Saposnik G, Maurino J, Saizar R, Bueri JA (2004). Undulating toe movements in brain death. Eur J Neurol.

[14] Conci F, Procaccio F, Arosio M, Boselli L (1986). Viscero-somatic and viscero-visceral reflexes in brain death. J Neurol Neurosurg Psychiatry.

[15] Araullo ML, Frank JI, Goldenberg FD, Rosengart AJ (2007). Transient bilateral finger tremor after brain death. Neurology.

[16] Jung KY, Han SG, Lee KH, Chung CS (2006). Repetitive leg movements mimicking periodic leg movement during sleep in a brain-dead patient. Eur J Neurol.

[17] Schneider H, Matakas F (1971). Pathological changes of the spinal cord after brain death. Acta Neuropathol.

[18] De Freitas GR, Lima MA, Andre C (2003). Complex spinal reflexes during transcranial Doppler ultrasound examination for the confirmation of brain death. Acta Neurol Scand.

[19] de Freitas GR, Andre C (2005). Absence of the Babinski sign in brain death: a prospective study of 144 cases. J Neurol.

[20] Han SG, Kim GM, Lee KH, Chung CS, Jung KY (2006). Reflex movements in patients with brain death: a prospective study in a tertiary medical center. J Korean Med Sci.

